# Profiling *Myxococcus xanthus* Swarming Phenotypes through Mutation and Environmental Variation

**DOI:** 10.1128/JB.00306-21

**Published:** 2021-11-05

**Authors:** Linnea J. Ritchie, Erin R. Curtis, Kimberly A. Murphy, Roy D. Welch

**Affiliations:** a Department of Biology, Syracuse University, Syracuse, New York, USA; b Department of Biology, Augustana College, Rock Island, Illinois, USA; Geisel School of Medicine at Dartmouth

**Keywords:** *Myxococcus xanthus*, biofilm, flares, genotype, microscopy, motility, phenotype, predation, ripples, swarm

## Abstract

Myxococcus xanthus is a bacterium that lives on surfaces as a predatory biofilm called a swarm. As a growing swarm feeds on prey and expands, it displays dynamic multicellular patterns such as traveling waves called ripples and branching protrusions called flares. The rate at which a swarm expands across a surface, and the emergence of the coexisting patterns, are all controlled through coordinated cell movement. M. xanthus cells move using two motility systems known as adventurous (A) and social (S). Both are involved in swarm expansion and pattern formation. In this study, we describe a set of M. xanthus swarming genotype-to-phenotype associations that include both genetic and environmental perturbations. We identified new features of the swarming phenotype, recorded and measured swarm expansion using time-lapse microscopy, and compared the impact of mutations on different surfaces. These observations and analyses have increased our ability to discriminate between swarming phenotypes and provided context that allows us to identify some phenotypes as improbable outliers within the M. xanthus swarming phenome.

**IMPORTANCE** Myxococcus xanthus grows on surfaces as a predatory biofilm called a swarm. In nature, a feeding swarm expands by moving over and consuming prey bacteria. In the laboratory, a swarm is created by spotting cell suspension onto nutrient agar in lieu of prey. The suspended cells quickly settle on the surface as the liquid is absorbed into the agar, and the new swarm then expands radially. An assay that measures the expansion rate of a swarm of mutant cells is the first, and sometimes only, measurement used to decide whether a particular mutation impacts swarm motility. We have broadened the scope of this assay by increasing the accuracy of measurements and introducing prey, resulting in new identifiable and quantifiable features that can be used to improve genotype-to-phenotype associations.

## INTRODUCTION

Myxococcus xanthus is a Gram-negative bacterium that moves across surfaces as a motile biofilm called a swarm. In the laboratory, swarm movement is almost always studied on an agar surface. When several million M. xanthus cells in a few microliters of liquid suspension are spotted onto a nutrient agar plate, the liquid is absorbed into the agar within a few minutes, leaving the cells settled on the surface in the shape of a circle. After some time, the cells begin to move, and the circle becomes a swarm. Under nutrient-rich conditions, a swarm will expand outward in all directions and double or triple in diameter within a few days (1–3).

Swarming M. xanthus cells move using two mechanistically distinct and genetically separable molecular motors. These drive two different types of surface movement, called adventurous (A) motility and social (S) motility, respectively (4, 5). Cells lacking both forms of motility do not move on agar, and swarms of such cells do not expand. The A motor that drives A motility functions within a cell by forming points of adhesion to an external surface such as agar and then rolling the cell like a tank tread or twisting it like a threaded screw (6–8). The S motor that drives S motility functions by extending a fiber called a pilus from one end of the cell, tethering the pilus to an external object, and then retracting it like a winch (9–12). The A and S motors differ in that the A motor seems to involve a pushing force, whereas the S motor involves a pulling force. Mutant swarms possessing only the A motor (A^+^ S^−^) expand faster on harder agar than swarms possessing only the S motor (A^−^ S^+^), whereas the opposite is true on softer agar (4, 5, 13). Perhaps hard agar better allows the A motility-associated adhesion points to attach and roll or twist a cell forward, while the lower drag coefficient of soft agar facilitates S motility-associated pili winching a cell forward. Both A and S motors periodically reverse by swapping the leading and lagging poles of each rod-shaped cell, thereby causing the cell to move in the opposite direction.

This mechanistic description of how the A and S motors interact with an agar surface provides a framework for the interpretation of new data regarding the impact of mutation on swarm motility. Specifically, the expansion rate on agar of a swarm of mutant cells (which we refer to as a mutant swarm) is compared to the wild type (WT) to measure the mutation’s phenotypic impact with respect to motility. Further, a comparison of that swarm’s relative expansion rates on hard and soft agar provides an indication of how much A and S motility, respectively, is involved in the phenotype. These data and their interpretation form the rationale behind the 72-hour swarm expansion (72H) assay (13, 14). The 72H assay is simple and widely used, with some minor variations between laboratories: spot mutant cells from a shaking liquid culture onto 0.4% (soft), 1.0%, and 1.5% (hard) agar nutrient plates; allow cells to settle as circular swarms; measure initial swarm diameters; incubate at ∼30°C for 72 h; measure final swarm diameters; and report the difference between final and initial diameters as each mutant strain’s “swarm expansion rate.” The expansion rates of mutant swarms on hard and soft agar compared to the WT serve as proxies for how much A and S motility, respectively, have been affected by the mutation.

The 72H assay is only one of several ways to measure the impact of mutation on motility in M. xanthus, and by themselves, a mutant swarm’s hard and soft agar expansion rates are never considered definitive in determining if a mutated gene is involved in either A or S motility. The 72H assay is the most scalable of the common M. xanthus motility assays, however, and it is therefore almost always the first, and often the only, motility data available when making large-scale comparisons of many mutant swarms.

This presents a problem when considering the biological interpretation of 72H data in isolation, because the set of genes involved in motility is only a subset of the larger set of genes whose mutation could impact the results of the 72H assay for any reason. Genes that could impact the results of the 72H assay include not only those involved directly in the A and S systems but also any genes involved more generally in cell motility and its regulation, as well as other genes that may have only a pleiotropic effect on a swarm’s expansion rate. Any large-scale interpretation of multiple mutant swarms’ 72H data in isolation must assume that the mutated genes could be any of these. Herein lies the problem: although the genes directly involved in the A and S systems may be distinct and separable, the larger set of genes that impact swarm expansion may not be. With only 72H data, it is impossible to determine whether the mutation was in a gene directly involved in A or S motility or in another unrelated gene. One solution is to refrain from making any biological interpretation based on 72H data alone. This does not diminish the utility of the 72H assay in making genotype-to-phenotype associations. In fact, dropping the need for a biological interpretation opens the door to test protocol modifications and examine new phenotypic features for no reason other than to improve the ability to discriminate between mutant swarms.

Standard laboratory conditions for protocols like the 72H assay involve a smooth agar surface and Casitone as a nutrient source. These conditions are very different from those found in nature, where M. xanthus evolved to swarm across a range of surface types and consume a wide variety of prey bacteria. Therefore, the phenotypic impact of a mutation may not manifest under the deliberately simple and stable laboratory conditions of a 72H assay, its impact may be subtle, or it may involve changes to features of the M. xanthus swarm phenotype that seem irrelevant. For example, traveling waves of cell density, called ripples, are sometimes a feature of an M. xanthus swarm, and multicellular projections, called flares, are sometimes a feature of a swarm’s edge (4, 5, 15–19). Like swarm expansion, ripples and flares also require cell movement, but they have never been included as part of a swarm expansion assay.

We have observed and characterized the M. xanthus swarming phenotype beyond the experimental conditions of the 72H assay. By exploring new surfaces, nutrient sources, and data recording methods, we identified novel, reproducible, and quantifiable phenotypic features that can be applied to genome annotation and genotype-to-phenotype mapping. We then tested how these features could be used to distinguish differences between the WT and the phenotypes of 50 single-gene insertion-disruption mutant strains.

## RESULTS

### Wild-type swarming varies according to assay and surface type.

Swarm expansion assays were performed using seven experimental protocols. Four surfaces were used: 0.4% agar (0.4%), 1.0% agar (1.0%), 1.5% agar (1.5%), and a lawn of Escherichia coli spotted onto 1.5% agar (Prey). Two assay protocols were used to record and measure data, which we refer to as 25H and 72H conditions (see Materials and Methods). The final measurement under 25H conditions was made 1 h longer than the first measurement under 72H conditions so that the five intervals of 25H conditions would be equal. Both 25H and 72H swarm expansion data are reported as velocity in millimeters per hour. All four surfaces were used under both conditions with one exception; the combination of Prey under 72H conditions (Prey/72H) was not performed because the WT swarm expanded rapidly enough that it would always expand beyond the boundary of the Prey spot well before the end of the assay. Therefore, 72H conditions were deemed too long for the Prey surface.

**FIG 1:**
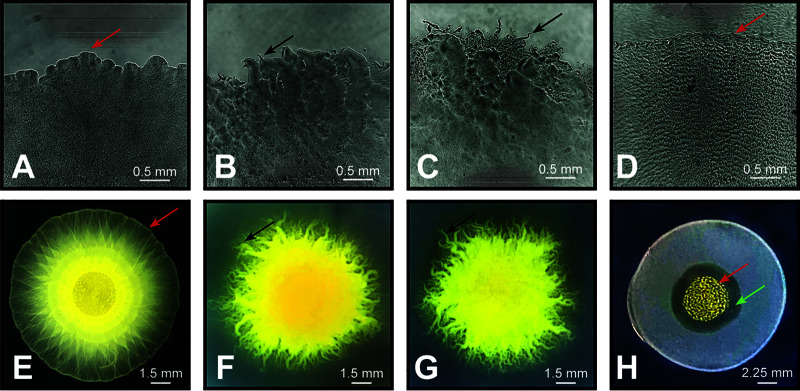
Wild-type M. xanthus swarm expansion on different surfaces. WT M. xanthus swarms have different phenotypes on different surfaces: 0.4% agar (A and E), 1.0% agar (B and F), 1.5% agar (C and G), and prey on 1.5% agar (D and H). Top-row images (A to D) were the last in a time-lapse image stack taken during a 25H assay (bar, 0.5 mm). Image H was also taken after 25 h of growth (bar, 2.25 mm), and images E to G were taken after 72 h of growth (bar, 1.5 mm). Red arrows mark the smooth swarm edges observed on 0.4% agar and prey. Black arrows mark the flares observed at the edges of swarms on 1.0% and 1.5% agar. Green arrow marks the zone of predation observed between the M. xanthus spot and prey E. coli.

WT swarms expand radially from the point of inoculation on all surfaces under both conditions. Flares were observed at the edge of swarms only on 1.0% and 1.5% (Fig. 1B, C, F, and G, black arrows), while on 0.4% and Prey, WT swarms had a mostly smooth edge (Fig. 1A, D, E, and H, red arrows). Ripples were observed only on Prey (Fig. 1D), appearing throughout the swarm within the first few hours and continuing until the end of the Prey/25H assay. An M. xanthus swarm on Prey also forms a cell-free “zone of predation,” which appears within the first few hours between the advancing edge of the M. xanthus swarm and the receding edge of the Prey lawn (Fig. 1H, green arrow).

**FIG 2:**
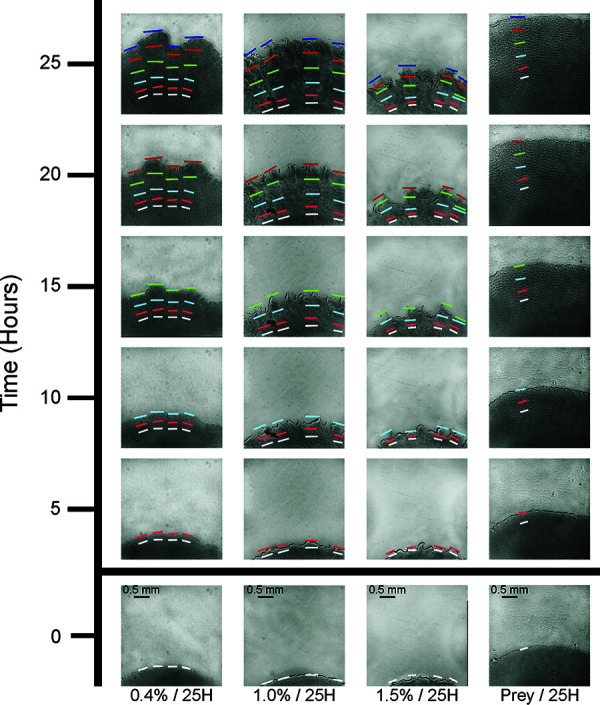
Progression of wild-type M. xanthus swarm expansion on different surfaces (25H). WT swarm expansion tracked from different positions on the swarm edge. Columns represent 5-h time points for each of the surfaces used in this study. From bottom, left to right: 0.4%/25H, 1.0%/25H, 1.5%/25H, and Prey/25H. Colored bars mark the time point measurements (white, 0 h; pink, 5 h; light blue, 10 h; green, 15 h; red, 20 h; navy, 25 h). Scale bar in the 0HR images (bottom row) applies to every image.

Images from time-lapse microscopy were used to measure swarm expansion at 5-h intervals. Figure 2 shows the progression of a WT swarm on all four surfaces under 25H conditions. To determine the swarm expansion rate on 0.4%, 1.0%, and 1.5% agar, the position of the swarm edge was measured at four locations within the field of view (Fig. 2, first three columns from left). When flares were present on 1.0% and 1.5%, which typically happened at later intervals, measurements were taken at the tip of the furthest flare at each location (for example, 1.0% and 1.5% in Fig. 2). Using four locations for each time point made it possible to account for the swarm edge irregularity caused by the flares. The swarm edge on Prey was the exception. There were almost no edge irregularities at any time point, and there was little variation between measurements. Therefore, a single measurement was used to represent swarm expansion on Prey. Prey/25H assays also were started with more of the swarm showing within the initial field of view, so that the appearance of ripples within the swarm could be observed and recorded.

### Mutant strains respond differently to changes in assay conditions.

**FIG 3:**
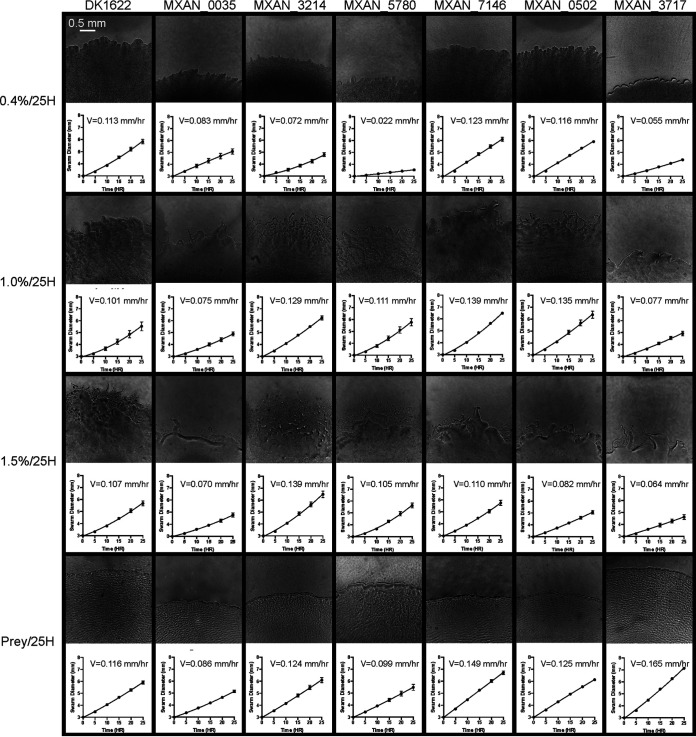
Motility phenotypes displayed in 25H assays. Swarm expansion tracked on all surfaces under 25H assay conditions. Columns are WT (left) and phenotypically representative mutant strains. Rows are for each surface. From the top, 0.4%, 1.0%, 1.5%, and Prey. Images are from the end of an assay. Graphs show expansion at 5-h intervals and represent at least three replicates. Velocity is at the top of each graph. The scale bar (top left) is for all images.

Graphs representing WT 25H expansion rates on all four surfaces are presented in Fig. 3, together with calculated velocities and representative swarm images (Fig. 3, first column on left). The same set of four 25H assays were also performed on the three historic reference strains DK1218, DK1253, and DK11316, as well as the 50 single-gene insertion-disruption mutant strains listed in Table 1. The list of genes includes 34 transcriptional regulators from three major families (ECF sigma factors, one-component regulators, and NtrC-like activators) and 16 from the ABC transporter family. These four gene families are well represented in the M. xanthus genome; 36 of the genes have been discussed in prior publications regarding the phenotypic impact of their mutation in M. xanthus, and 13 have a common name (references and common names are included in Table 1). This assortment of unknown and previously studied regulatory genes was chosen because their disruption was deemed likely to produce a range of motility phenotypes. They have not been previously studied as a set.

**TABLE 1:** M. xanthus wild-type and mutant strains used in this study

Strain	Gene name(s)	Function	Reference or source
Myxococcus xanthus (DK1622)		Wild type	4, 5
Mxan_0035		ABC transporters	31
Mxan_0079		One component	This study
Mxan_0172		Ntr_C like activators	32
Mxan_0180		Ntr_C like activators	33
Mxan_0213		One component	This study
Mxan_0311		One component	This study
Mxan_0502		One component	This study
Mxan_0603		Ntr_C like activators	32
Mxan_0622		ABC transporters	31
Mxan_0665		One component	This study
Mxan_0681		ECF	34
Mxan_0685		ABC transporters	31
Mxan_0721		ABC transporters	31
Mxan_1078	*spdR* (*nla19*)	Ntr_C like activators	35
Mxan_1128	*frgC*	Ntr_C like activators	36
Mxan_1153	*sufB*	ABC transporters	31
Mxan_1167	*nla28*	Ntr_C like activators	23
Mxan_1245	*sasR*	Ntr_C like activators	37, 38
Mxan_1565		Ntr_C like activators	32
Mxan_2030		ECF	34
Mxan_2268		ABC transporters	31
Mxan_2711		One component	This study
Mxan_3214	*actB*	Ntr_C like activators	39
Mxan_3702		One component	This study
Mxan_3717		ABC transporters	31
Mxan_3718		ABC transporters	31
Mxan_3719		ABC transporters	31
Mxan_4196		Ntr_C like activators	32
Mxan_5101		ECF	34
Mxan_5124	*mrpB*	Ntr_C like activators	40, 41
Mxan_5153	*crdA*	Ntr_C like activators	42
Mxan_5263		ECF	34
Mxan_5410		ECF	34
Mxan_5680		Ntr_C like activators	This study
Mxan_5747		ABC transporters	31
Mxan_5777	*pilR2* (*nla23*)	Ntr_C like activators	23, 43, 44
Mxan_5780	*pilI*	ABC transporters	23, 45
Mxan_5781	*pilH*	ABC transporters	23, 45
Mxan_5879		Ntr_C like activators	32
Mxan_5894		One component	This study
Mxan_6173		ECF	34
Mxan_6426		Ntr_C like activators	This study
Mxan_6569		ABC transporters	31
Mxan_6575		ABC transporters	31
Mxan_6827	*natA*	ABC transporters	31
Mxan_6889		One component	46
Mxan_7143		Ntr_C like activators	32
Mxan_7146		ABC transporters	31
Mxan_7214		ECF	34
Mxan_7440	*epsI* (*nla24*)	Ntr_C like activators	23 – 25
M. xanthus (DK1218)		A^−^ S+	4
M. xanthus (DK1253)		A^+^ S^−^	4
M. xanthus (DK11316)		A^−^ S^−^	22

While most mutant strains’ swarm expansion rates were similar to that of the WT, some swarmed slower than the WT on all surfaces. For example, Mxan_0035 (Fig. 3, first column from left) was always slower than the WT. Nevertheless, it produced flares on both 1.0% and 1.5% and ripples on Prey, indicating that the cells were actively moving even though the swarm was expanding slower. While no strains were faster than the WT on all agar concentrations, several did swarm faster than the WT on at least two out of the three. For example, Mxan_3214 (*actB*) (Fig. 3, third column from left) swarmed faster than the WT on 1.0% and 1.5%. Some strains differed from the WT on only one concentration of agar. For example, Mxan_5780 (*pilI*) (Fig. 3, fourth column from left) was slower than the WT only on 0.4%, while Mxan_7146 (Fig. 3, fifth column from left) was faster only on 1.0% agar, although it was also faster on Prey. The rate of Mxan_0502 (Fig. 3, sixth column from left) displayed great variability with respect to its swarm expansion rate on different agar surfaces, expanding like the WT on 0.4% (and Prey), faster than the WT on 1.0%, and slower than the WT on 1.5%. Finally, several strains displayed a different phenotype on Prey than on any agar concentration. For example, Mxan_3717 (Fig. 3, seventh column from left) was slower than the WT on all three agar surfaces but faster than the WT on Prey.

**FIG 4:**
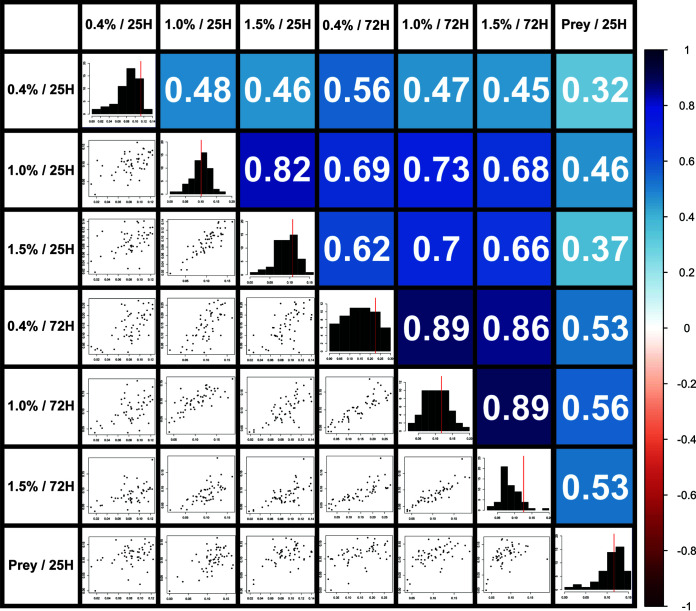
Pairwise correlations across assay conditions. (Left) Scatterplots of all 54 strains (50 mutants, WT, DK1218, DK1253, and DK11316) display comparisons of swarm expansion rates (millimeters per hour) for all assay pairs. Histograms (center diagonal) of the distribution of swarm expansion rates for each assay (red line marks WT for each histogram). (Right) Numbers and colors are the Spearman’s rank correlation coefficient for each pairwise assay comparison. Colored bar (right) scales colors to values. File S3 has enlarged graphs.

72H assays were also performed on all three agar surfaces for the WT, the three historic reference strains DK1218, DK1253, and DK11316, and the 50 mutant strains, and pairwise comparisons were made for each combination of all seven types of assays. A multiple Spearman correlation graph (20) showing each pairwise combination revealed positive correlations in every case (Fig. 4), although the degree of correlation varied between surfaces (0.4%, 1.0%, 1.5%, and Prey) and conditions (25H and 72H). While discussing the interpretation of correlation data, the following rule-of-thumb value range assignments will be applied: 0.9 to 1.0, very high; 0.7 to 0.9, high; 0.5 to 0.7, moderate; 0.3 to 0.5, low; 0.0 to 0.3, negligible (21). These same range assignments would also apply to negative correlations (0.0 to −1.0), but as previously stated, no negative correlations were observed. Although cutoffs for these range assignments may seem arbitrary, they are a commonly used frame of reference for discussing coefficient analysis. Most notably, there were high correlations between the three agar surfaces under 72H conditions (0.86, 0.89, and 0.89). These correlations were unexpectedly high because hard and soft agars are meant to distinguish between motility mutants, and this high degree of correlation would seem to diminish any discriminatory power. This apparent inconsistency will be addressed in Discussion. Agar surface correlations under 25H conditions were closer to what was expected, being high for 1.0% compared to 1.5% (0.82) but only moderate for 0.4% compared to 1.0% (0.48) and 0.4% compared to 1.5% (0.46). Correlation between 25H and 72H conditions for the same agar surface was high for 1.0% (0.73) but only moderate for 0.4% and 1.5% (0.56, 0.66). These correlations were unexpectedly low, since 25H and 72H conditions represent nothing more than two ways of observing and measuring the same swarm expansion phenomenon. This apparent inconsistency will also be addressed in Discussion.

Swarm expansion on Prey did not show high correlation with any agar surface using either condition. Correlation was low between Prey and all agar surfaces using 25H conditions (0.32, 0.46, and 0.37) and moderate between Prey and all agar surfaces using 72H conditions (0.53, 0.56, and 0.53).

### Collective mutant behavior provides insight and reveals new phenotypes.

**FIG 5:**
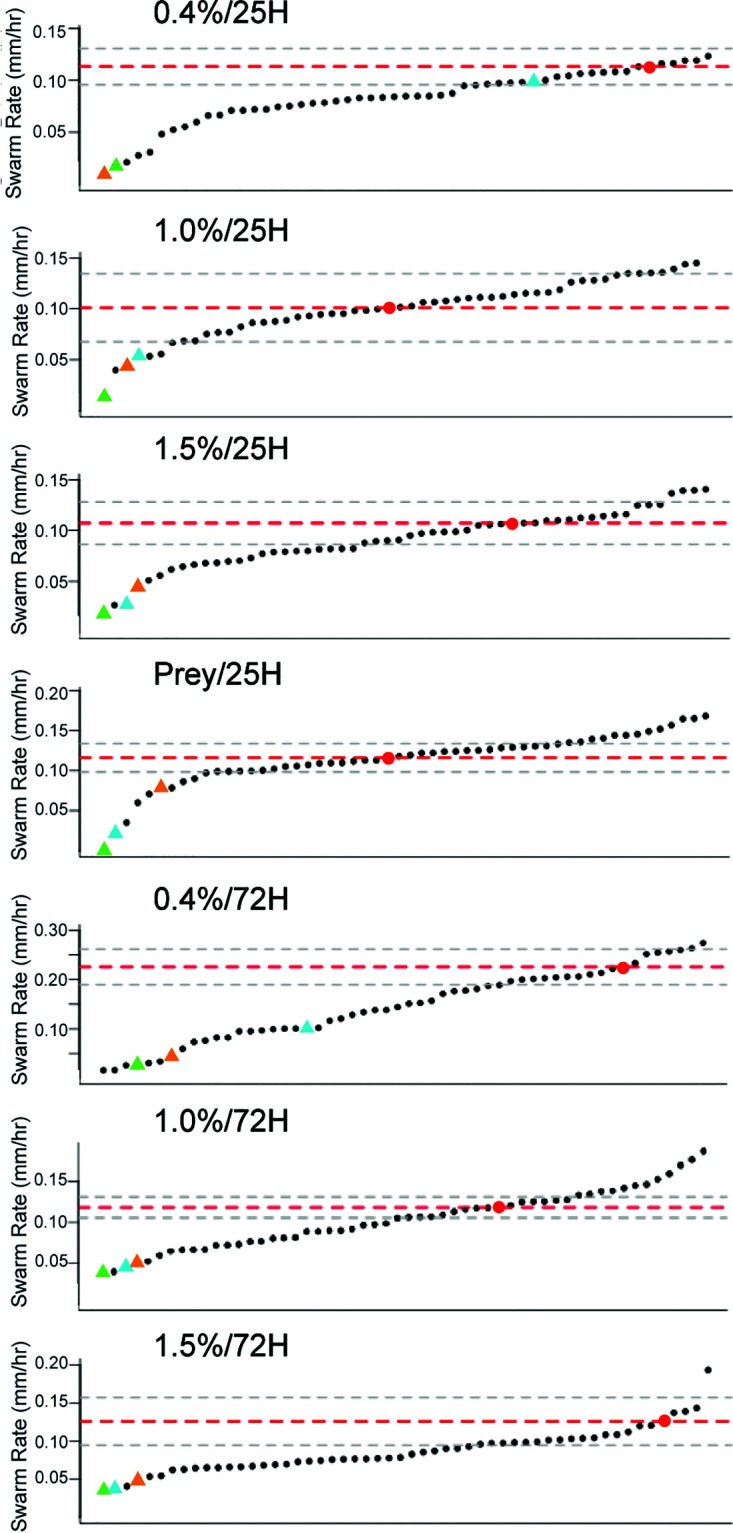
Wild-type and mutant M. xanthus swarm expansion rates for all assay conditions. Graphs display the swarm expansion rates (millimeters per hour) of 54 strains (50 mutants, WT, DK1218, DK1253, and DK11316) arranged from slowest (left) to fastest (right). The expansion rate of the WT (red dots, red dotted lines) is marked together with one standard deviation (gray dotted lines). Historical reference strains are marked as colored triangles: green, DK11316; blue, DK1218; orange, DK1253. The rates of all mutant strains are provided in Table S1.

Expansion rates for the WT, the historic reference strains, and the 50 mutant strains were combined and displayed in increasing order according to their expansion rates for all seven assays (Fig. 5). The graphs have at least three notable features: (i) all seven assays display a continuous distribution of swarm expansion rates; (ii) the WT swarm expansion rate is either near the mean (1.0%/25H, Prey/25H), faster than the mean (1.5%/25H, 1.0%/72H), or among the fastest (0.4%/25H, 0.4%/72H, 1.5%/72H) for each assay; and (iii) the fastest swarm expansion rate for 0.4%/25H is near the mean expansion rate for 0.4%/72H. All swarm expansion rates for all mutant strains are listed in File S1 in the supplemental material, and all raw data contributing to these rates are in File S2.

The three historic reference strains (DK1218, DK1253, and DK11316) were included in this study as controls to confirm that the results of these assays match those of prior work, up to and including the first published reports linking agar concentration to A and S motility (4, 5, 13). DK1218 (*cglB2*) is defective in A motility (A^−^ S^+^), DK1253 (*tgl-1*) is defective in S motility (A^+^ S^−^) (4, 5), and DK11316 is nonmotile (A^−^ S^−^) (*pilA*::Tc^r^ Δ*cglB*) (22). All three strains expanded more slowly than the WT on all surfaces using both 25H and 72H conditions, and, as expected, DK11316 showed almost no expansion. The expansion rate of DK1218 was faster than that of DK1253 on 0.4% and DK1253 was faster than DK1218 on 1.5%, although DK1253 is much slower than the WT on both 0.4% and 1.5% agar. All of these rates, including the relatively slow expansion of DK1253 on 1.5% agar, are in good agreement with prior published results, as Shi et al. also found that DK1253 was relatively slow on both hard and soft agar (13). Only DK1218 formed flares, and only on 1.0% and 1.5%, and none of these reference control strains formed ripples on Prey. Perhaps some degree of both A and S motility is required for ripples to manifest.

**FIG 6:**
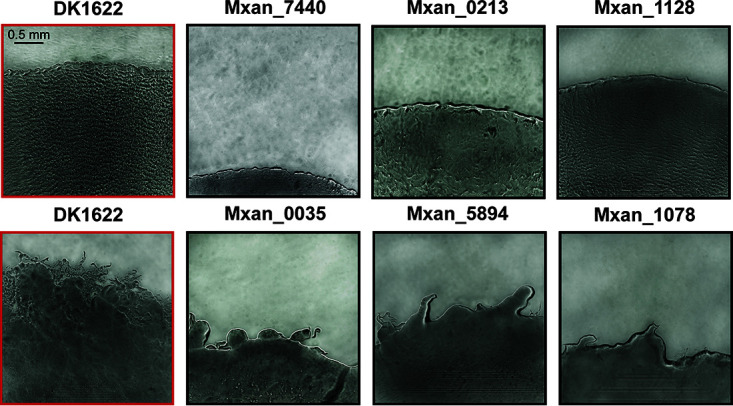
Qualitative differences in swarm expansion phenotypes. Representative mutant swarm phenotypes that are qualitatively different from the WT. Images show swarm edges on Prey/25H (top row) or 1.5%/25H (bottom row). DK1622 (WT) (left, red boxes) is provided as a reference. Mxan_7440 displayed no ripples during the Prey/25H assay. Mxan_0213 appeared to have ripples with a wavelength longer than WT, and Mxan_1128 displayed ripples that appeared to move more slowly. Mxan_0035, Mxan_5894, and Mxan_1078 all produced a smaller number of flares that are noticeably different from the WT when plated on 1.5% agar. The scale bar in the top left image applies to every image in the figure.

Time-lapse image data from the WT and the 50 mutant strains also have several notable features regarding ripples and flares. Rippling on Prey proved to be a robust phenotype, observed in 49 of the 50 mutant strains. The only strain that appeared not to ripple, Mxan_7440 (*nla24*), showed little swarm expansion on any surface using either condition, which agreed with findings from previous studies (23–25). This does not mean that a nonrippling phenotype is linked to a low rate of swarm expansion, as several strains that had low expansion rates similar to Mxan_7440 did ripple on Prey (e.g., Mxan_6827, Mxan_7143, Mxan_0035, and Mxan_1078). The ripples of several mutant strains appeared noticeably different from the WT with respect to their amplitude, speed, wavelength, frequency, and other discernible features (Fig. 6, top row). For example, a swarm of Mxan_0213, despite expanding faster than the WT on Prey, appeared to produce smaller, slower ripples. A swarm of Mxan_1128 also appeared to have slower ripples throughout predation, despite having an expansion rate similar to that of WT. These differences are often easier to discern when viewed as time-lapse movies, but attempts to quantify this observation were unsuccessful. Videos in Files S4 and S5 are included as examples.

Flares were observed in most mutant strains, although they proved to be less robust than ripples. Like ripples, the flares of several mutant strains appeared different from the WT with respect to discernible features, such as length, width, number, curvature, and branching (Fig. 6, bottom row). Some strains produced an irregular edge but with no distinct protrusions (i.e., flares), even on harder (1.0% and 1.5%) agar surfaces, and these were also distinguishable from the WT (e.g., Mxan_0035, Mxan_5894, and Mxan_1078). Their expansion rates were mostly slower than that of the WT, except for Mxan_5894 on 1.0% agar, which was similar to the WT. Again, attempts to quantify this observation were unsuccessful.

## DISCUSSION

An M. xanthus swarm’s expansion rate is a single measurement that does not fully represent the complicated swarming phenotype. Swarms expand at different rates on 0.4% and 1.5% agar, and they look different in other ways that are hard to characterize and quantify. These observations are true for both WT and mutant strains. By varying the laboratory environment beyond standard assay conditions and by collecting some additional data across a set of mutant strains, we were able to identify and characterize several new features of the M. xanthus swarming phenotype.

The 72H assays on 0.4%, 1.0%, and 1.5% are closest to a historically standard set of swarm expansion assay conditions. The relative expansion rates of the 50 mutant strains provide additional information about the WT phenotype. The WT was close to the fastest on 0.4% agar, but on 1.0% and 1.5% it was nearer the mean. This observation is important because it indicates the probability of any future mutant strains being faster or slower than the WT on different agar concentrations. On 0.4% it is much more likely that mutant strains will be slower than the WT, but on 1.0% or 1.5% they could be slower or faster with roughly equal probability. If we hypothesize that the continuous distributions of expansion rates for the WT and these 50 mutant strains under 72H conditions represent an approximation of the distributions for all mutant strains under 72H conditions, then it would be reasonable to postulate that a fast expansion rate is not a trait that has been optimized in the WT for higher agar concentrations.

While it was not surprising that the multiple Spearman’s rank correlation values were all positive, since cell movement is part of every assay, the results of some comparisons were unexpected in their degree of correlation. For example, given the long-held understanding that soft agar better facilitates S motility and hard agar better facilitates A motility, the expectation was that there would be little positive correlation between any of the different agar concentrations for a given assay. For example, a low to negligible positive correlation range between 0.4%/72H and 1.5%/72H was expected, but the opposite was observed, with the correlation value (0.86) being in the high range assignment. Indeed, all three agar concentrations tested under the 72H conditions showed surprisingly high degrees of positive correlation. This might be expected if very few mutant strains differed from the WT with respect to their swarm expansion rate, but this also was not observed.

Perhaps these high correlations reveal something about the relative size and relationship between the set of genes whose mutation impacts swarm expansion and the subset of those genes whose mutation directly impacts either A or S motility. Namely, if the genes that directly impact A or S motility represent only a small subset of the genes that impact swarm expansion, then, for any random set of mutant strains, the majority exhibiting a swarm expansion phenotype different from the WT would not specifically target either A or S motility or swarm expansion on either hard or soft agar. This would explain why the correlation coefficient between 0.4%/72H and 1.5%/72H is high for a random set of 50 mutant strains. However, it would in no way diminish the importance of the swarm expansion assay as a means of discerning the relative impact of mutation on A and S motility for those strains whose mutation specifically targets either system.

A second unexpected result was that the high degree of positive correlation between 0.4%, 1.0%, and 1.5% agar was largely limited to 72H conditions, as the three agar concentrations displayed mostly moderate correlation coefficients under 25H conditions. A high degree of correlation was expected between 25H and 72H assay conditions for all agar concentrations. After all, both conditions are presumably measuring the same swarm expansion phenotype, only in different ways. If the three agar concentrations display a high degree of correlation under 72H conditions, they should display the same degree of correlation under 25H conditions. This was not what was observed; instead, the correlation values between 25H and 72H conditions for 0.4% and 1.5% agar fell into the moderate range assignment, and only 1.0% were in the high range.

A plausible explanation for this discrepancy is that for the WT and many of the mutant strains, swarm expansion does not occur at a constant rate. A comparison of the distributions for 0.4%/25H and 0.4%/72H plots in Fig. 5 reveals that enough strains accelerate on 0.4% agar between 25 and 72 h that it forces a change in scale on the *y* axis between the plots. Examples of accelerating strains are Mxan_0172, Mxan_0213, Mxan_0665, Mxan_5101, Mxan_5780, and Mxan_6575. A few strains also seem to decelerate on 0.4% agar between 25 and 72 h, such as Mxan_0685 and Mxan_1245. Acceleration and deceleration are also not limited to 0.4% agar. One notable strain on 1.5% agar, Mxan_0172, accelerates from slower to much faster than the WT between 25 and 72 h. On the 1.5%/72H plot, Mxan_0172 is by far the fastest strain, appearing as the furthest outlier on the right side of the distribution with a velocity of nearly 0.2 mm/h. Velocities for each strain under 25H and 72H conditions are included in File S1 in the supplemental material.

Why would swarm expansion rates change between 25 and 72 h so that 72H data would display a higher degree of positive correlation than 25H data for all three agar concentrations? At this point, hypotheses that attempt to explain the biological underpinnings of this phenomenon must be based on scant evidence. Perhaps some accelerating mutant strains initially synthesize the exopolysaccharides that make up the matrix of the swarm more slowly than the WT, and this causes motility to be delayed. Perhaps some decelerating mutant strains have growth and division rates that are slower than those of the WT so that cell number cannot keep up with the initial rate of swarm expansion. Regardless of the underlying mechanism, it is reasonable to hypothesize that the manifestation of mutant phenotypes might require more than 25 h on some surfaces but that 72 h is sufficient for mutant swarm expansion phenotypes to manifest on all surfaces. If true, this would explain why only the 72H data display a high degree of positive correlation.

Admittedly, the previous paragraph is full of speculation. All or none of these hypotheses might prove to be correct, but determining the biological cause(s) of the mutant phenotypes characterized and quantified in this study is not its purpose. The purpose of this study is to observe, measure, and assess the discriminatory power of phenotypic features related to swarm motility so that more and better genotype-to-phenotype associations can be made. Data presented in this study show that a few minor modifications to the swarm expansion assay can provide greater discriminatory power because it can measure a larger set of reasonably independent variables that might be under genetic control.

From this perspective, Prey as a surface is more useful than hard or soft agar at discriminating between mutant swarm phenotypes because its correlation to any of the agar surfaces is only moderate. M. xanthus is a saprophytic predator; a swarm secretes enzymes and antibiotics to lyse prey extracellularly, and then it consumes the released cellular contents as it expands (26). This extracellular lysis can be observed under a microscope, where a small cell-free zone develops between the expanding outer edge of an M. xanthus swarm and the receding edge of the prey population (27). This zone of predation was observed on the WT and all mutant strains used in this study. It would therefore be reasonable to postulate that the surface on which the swarm is expanding is actually the 1.5% agar below the prey bacteria and not the bacterial mat itself, and that Prey represents a different nutrient source and not a different surface. This description may be a more accurate representation of the Prey swarm expansion assay, but M. xanthus swarms on Prey look very different from swarms on 1.5% agar. The expansion rate of a WT swarm on Prey is faster than that on 1.5%, and both WT and all mutant strains lack flares on Prey. In at least these ways, swarms on Prey appear closer to swarms on 0.4% agar than on 1.5% agar, and the nearly ubiquitous rippling observed on Prey makes the phenotype different from both.

Ripples and flares are both phenotypic features of swarm motility, but their purpose remains unknown. Flares clearly are not required to move quickly over surfaces, since swarm expansion on Prey and 0.4% agar was mostly faster than that on 1.0% or 1.5%, and no strains, including the WT, produced flares on either Prey or 0.4%. Ripples also have no obvious correlation to swarm expansion rates on Prey, since strains that expand slowly and strains that expand quickly all produced ripples (16, 19). Some mutant strains did produce ripples and flares that are discernably different from those of the WT, which indicates that these phenotypic features are under genetic control. This will make both ripples and flares more useful for genotype-to-phenotype association studies once they can be accurately quantified.

### Conclusions.

For a laboratory model organism with a reasonably long history of molecular genetic studies, such as M. xanthus, a set of standard phenotype assays emerges over time as a means of relating current experiments to prior work. The utility of these assays therefore depends on their reproducibility, and consistent protocols and analyses become established over time. Often a narrative evolves along with an assay, providing a biological explanation for phenotypic data. It can be useful to depart from these assays and narratives through naive genotype-to-phenotype association studies to provide a broader context and fresh perspective on the relationship between an organism’s phenotype and its species’ phenome, as we have done in this study of swarm motility. By tweaking the protocol and analysis of the standard 72-hour swarm expansion assay, we were able to observe and quantify more granular detail about the phenomenon of expansion, e.g., that expanding swarms can change their velocity, that a harder agar surface seems to favor the formation of flares, that rippling is a robust phenotype on Prey, and that Prey as a nutrient source changes the swarming phenotype in many ways beyond rippling. Although most of these findings appear tangential to the standard A and S motility narratives, they help us to better understand which phenotypes fall within “normal” parameters and which are outliers that may exist at the edge of the M. xanthus phenome.

## MATERIALS AND METHODS

### Bacterial strains and growth conditions.

WT M. xanthus (DK1622), the set of 50 single-gene insertion-disruption mutants, historic reference strains DK1218, DK1253, DK11316, and prey Escherichia coli (K-12) were used in this study (Table 1). WT M. xanthus was grown in CTTYE medium (1.0% Casitone, 0.5% yeast extract, 10 mM Tris-HCl [pH 8.0], 1 mM KH_2_PO_4_, and 8 mM MgSO_4_) or on petri dishes containing CTTYE broth and either 0.4%, 1.0%, or 1.5% agar. Both plates and liquid cultures were incubated at 32°C. Mutant insertion-disruption M. xanthus strains were grown in CTTYE supplemented with kanamycin (40 μg/ml) as a selective agent. E. coli was grown in LB broth (L3022; Sigma) and on LB broth with 1.5% agar. Predation assays were performed using TPM plates (10 mM Tris-HCl [pH 7.6], 1 mM KH_2_PO_4_, 8 mM MgSO_4_) with 1.5% agar.

### 25H motility assay.

Both WT M. xanthus and mutant strains were inoculated in CTTYE broth (40 μg/ml kanamycin added to mutant cultures) and incubated overnight at 32°C with agitation (∼300 rpm). Once cell density was ∼100 Klett (∼5 × 10^9^ cells/ml), cells were harvested and resuspended to 1,000 Klett in CTTYE. Five 2-μl spots were placed on CTTYE plates of agar concentrations 0.4% (soft), 1.0%, and 1.5% (hard). Plates were incubated at 32°C for 2 h before being inverted and placed on a three-dimensional (3D)-printed brightfield microscope (https://www.thingiverse.com/thing:2672065). The microscope was focused on the edge of one of the 2-μl spots so the edge of the spot took up less than 25% of the screen, leaving the rest of the field of view open in the direction that the spot would expand. One image was taken every 60 s for 25 h for a total of 1,500 images. These images were compiled into a time-lapse movie using Fiji (28) to show how the strain swarmed over 25 h.

### Prey assay.

All M. xanthus strains were prepared and incubated in the same manner as the 25H assay. Once cell density was ∼100 Klett (∼5 × 10^9^ cells/ml), cells were harvested, washed 2× in TPM broth, and resuspended to 1,000 Klett in TPM. E. coli cells were grown in LB broth at 32°C with agitation (∼300 rpm) and harvested at an optical density at 600 nm of ∼0.8. (∼6.4 × 10^8^ cells/ml). The E. coli cells were washed 2× in sterile autoclaved water and resuspended to the same concentration in autoclaved water, and 40 μl was spotted in the center of a TPM plate (1.5% agar). Once the spot dried, 2 μl of washed and resuspended M. xanthus cells was spotted directly on top of the dried E. coli spot. The plates were incubated at 32°C for 2 h before being inverted and placed on a 3D-printed bright-field microscope. The microscope was focused in the same manner as that for the 25H assay. One image was taken every 60 s for 25 h, and images were compiled into a time-lapse movie.

### 72H swarm expansion assay.

All M. xanthus strains were prepared, incubated, and harvested in the same manner as the 25H assay. Five 2-μl aliquots of 1,000 Klett M. xanthus cells were spotted on CTTYE plates of agar concentrations 0.4% (soft), 1.0%, and 1.5% (hard). Plates were incubated at 32°C for 72 h, and measurements of each spot were taken every 24 h using a ruler.

All motility assays were performed using bacteria under these growth conditions. Assays are named using the following convention: surface substrate (0.4%, 1.0%, or 1.5% agar, or Prey)/time and assay conditions (25H or 72H). For example, 0.4%/25H would refer to an assay performed on 0.4% agar under 25H conditions.

### Data availability.

Using time-lapse images, we marked the edge of each spot at times 0, 5, 10, 15, 20, and 25 h using Fiji (28). We measured the distance in pixels between the time points and converted pixels to millimeters. Because swarms expand radially and the initial swarm diameter created by the 2-μl spot was consistently 3 mm, we used each measurement to calculate overall swarm diameter at each time point: swarm diameter = (time point measurement [mm] × 2) + 3 mm. WT and mutant strains each had at least four replicates for each surface (0.4%, 1.0%, 1.5%, and Prey). Strains with swarm expansion rates of 80% or less than the WT rate on a surface are discussed as being slower than the WT, while strains with expansion rates at or above 120% of the WT rate are discussed as being faster than the WT. Correlation analysis, comparison, and visualization were done using R Studio (29), corrplot (20), ggplot2 (30), and Prism 9 version 4.00 for Windows (GraphPad Software, San Diego, CA, USA, www.graphpad.com). All data collected in this study are available in the supplemental material (see Tables S1 and S2 in the supplemental material).
