# Supplementary material for: Profiling Myxococcus xanthus Swarming Phenotypes through Mutation and Environmental Variation
Source: J Bacteriol. 2021 Nov 5;203(23):e00306-21. doi: 10.1128/JB.00306-21 (PMC8570273; doi:10.1128/JB.00306-21)
Supplement: Supplemental file 1 — Graphs. Download JB.00306-21-s0005.pdf, PDF file, 8.7 MB [file jb.00306-21-s0005.pdf]

Supplementary File S3 - Figure 4 Scatterplots & Histograms.

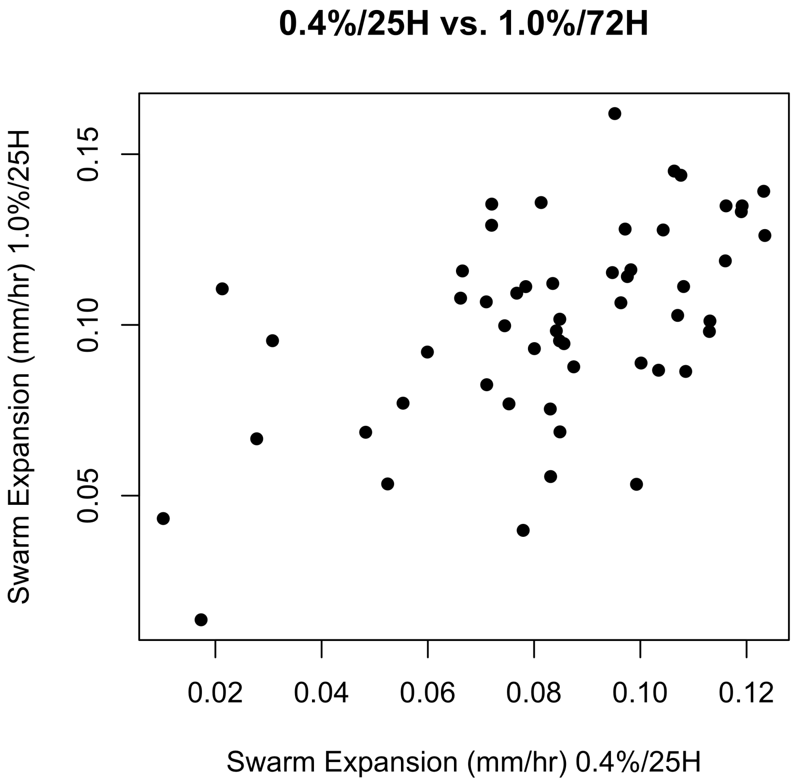

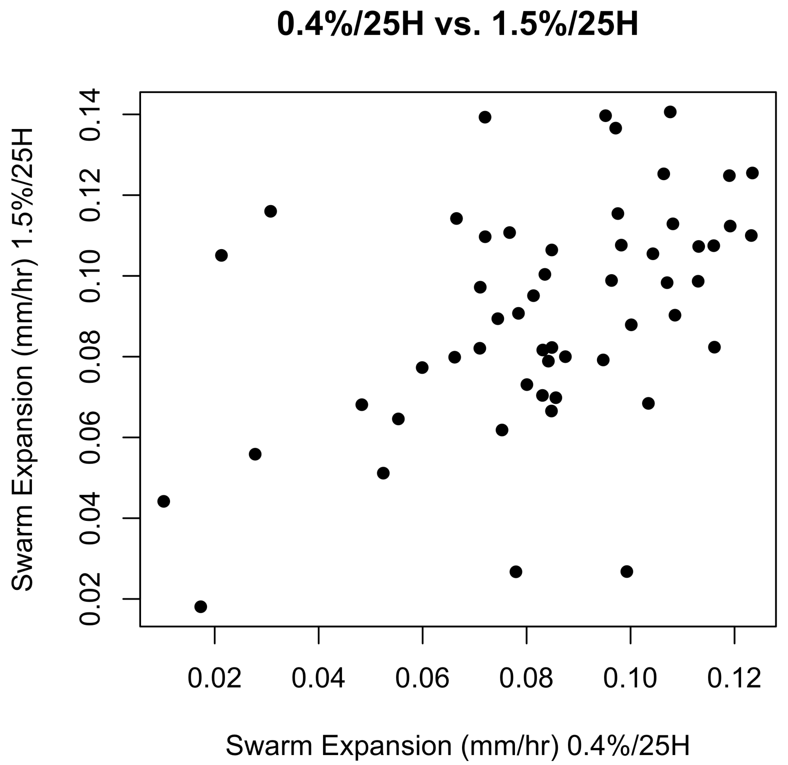

A

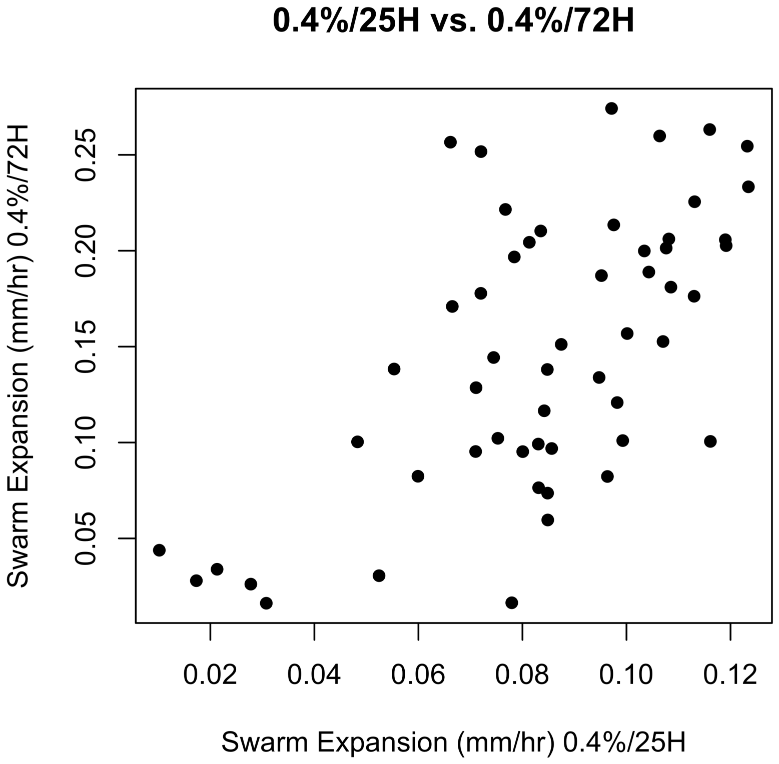
0.4%/25H vs. 0.4%/72H
Swarm Expansion (mm/hr) 0.4%/72H
0.05
0.10
0.15
0.20
0.25
0.02
0.04
0.06
0.08
0.10
0.12
Swarm Expansion (mm/hr) 0.4%/25H

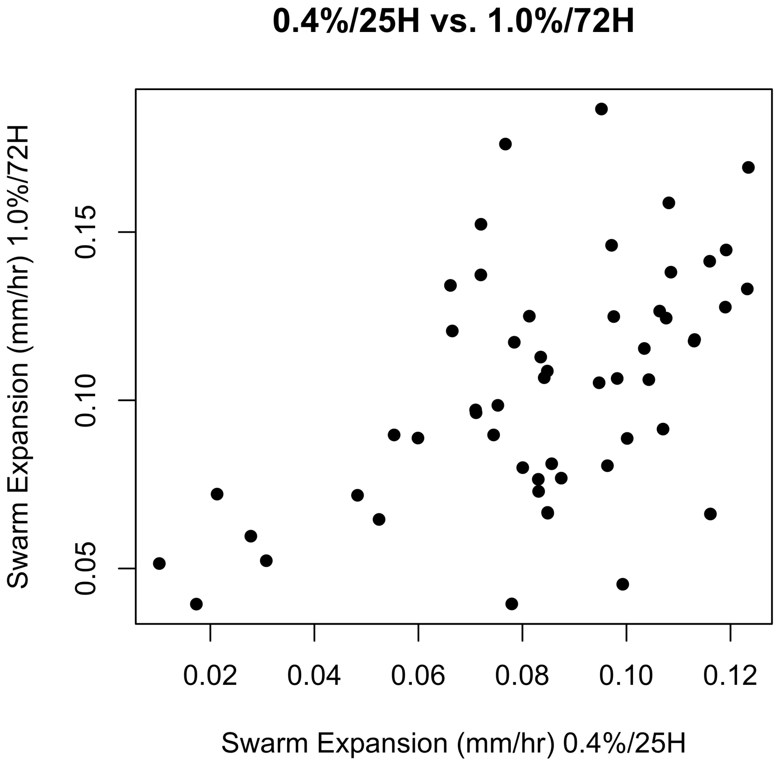
0.4%/25H vs. 1.0%/72H
Swarm Expansion (mm/hr) 1.0%/72H
0.05
0.10
0.15
0.02
0.04
0.06
0.08
0.10
0.12
Swarm Expansion (mm/hr) 0.4%/25H

B

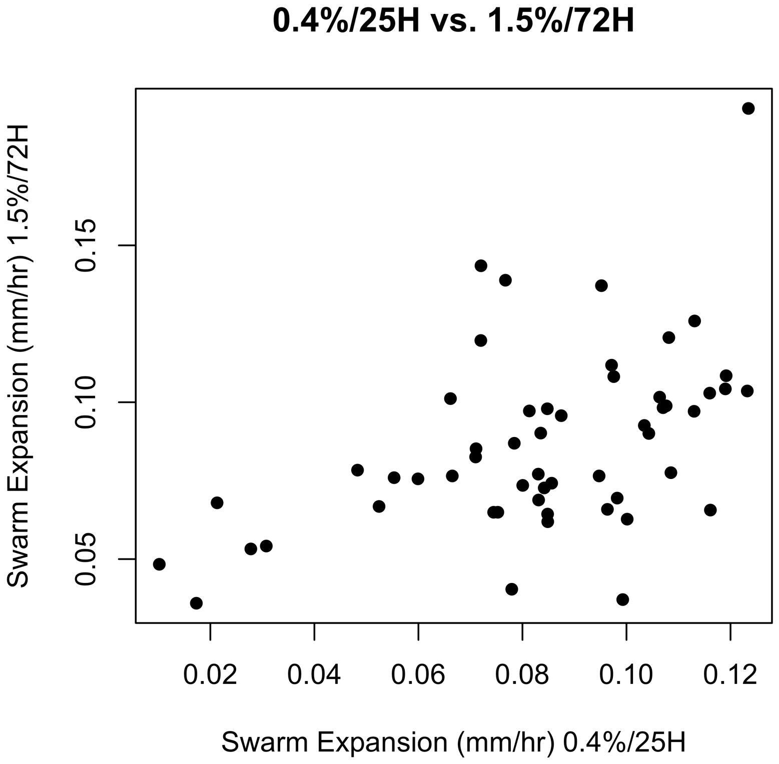
0.4%/25H vs. 1.5%/72H
Swarm Expansion (mm/hr) 1.5%/72H
0.05
0.10
0.15
0.02
0.04
0.06
0.08
0.10
0.12
Swarm Expansion (mm/hr) 0.4%/25H

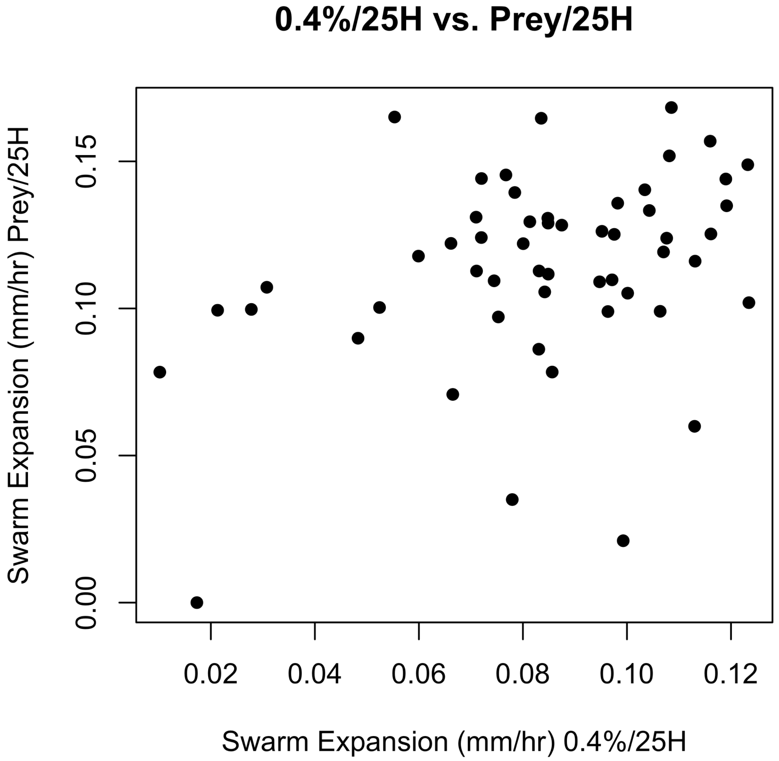
0.4%/25H vs. Prey/25H
Swarm Expansion (mm/hr) Prey/25H
0.00
0.05
0.10
0.15
0.02
0.04
0.06
0.08
0.10
0.12
Swarm Expansion (mm/hr) 0.4%/25H

C

1.0%/25H vs. 1.5%/25H

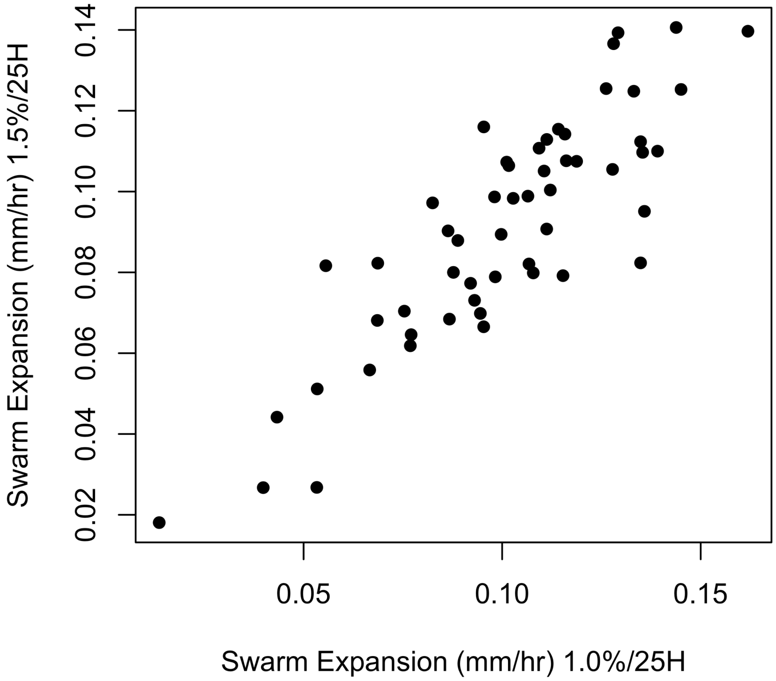

1.0%/25H vs. 0.4%/72H

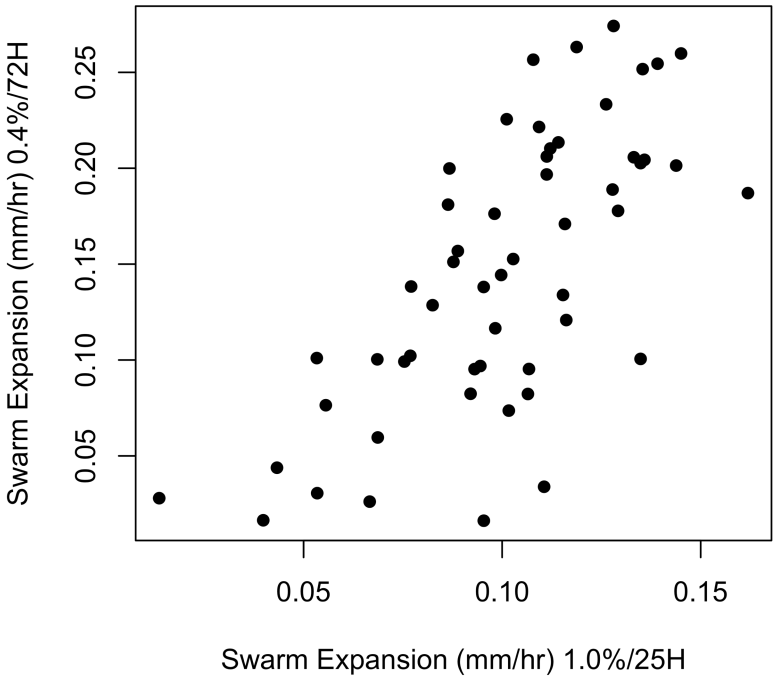

D

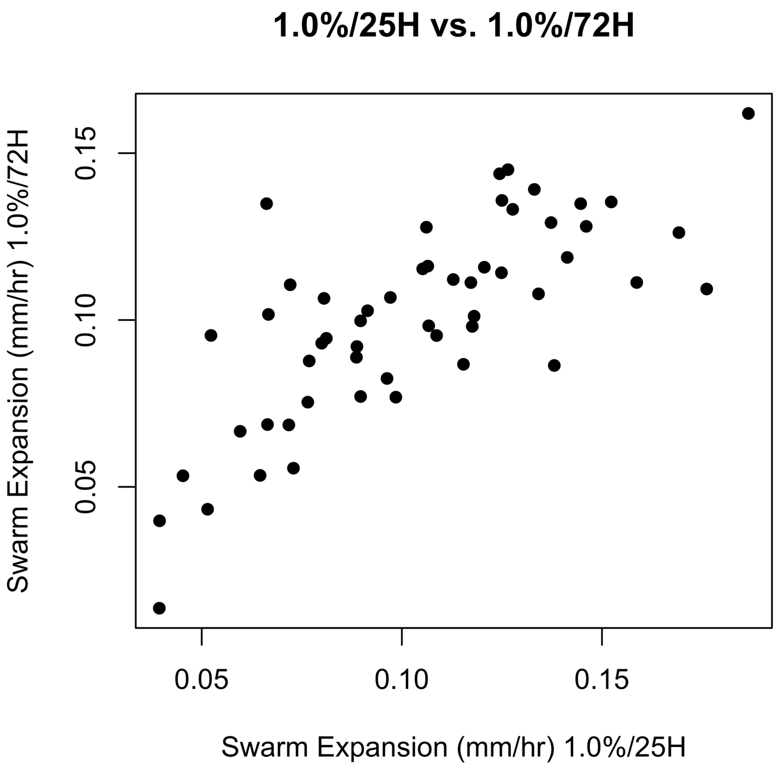
1.0%/25H vs. 1.0%/72H
Swarm Expansion (mm/hr) 1.0%/72H
0.05
0.10
0.15
Swarm Expansion (mm/hr) 1.0%/25H

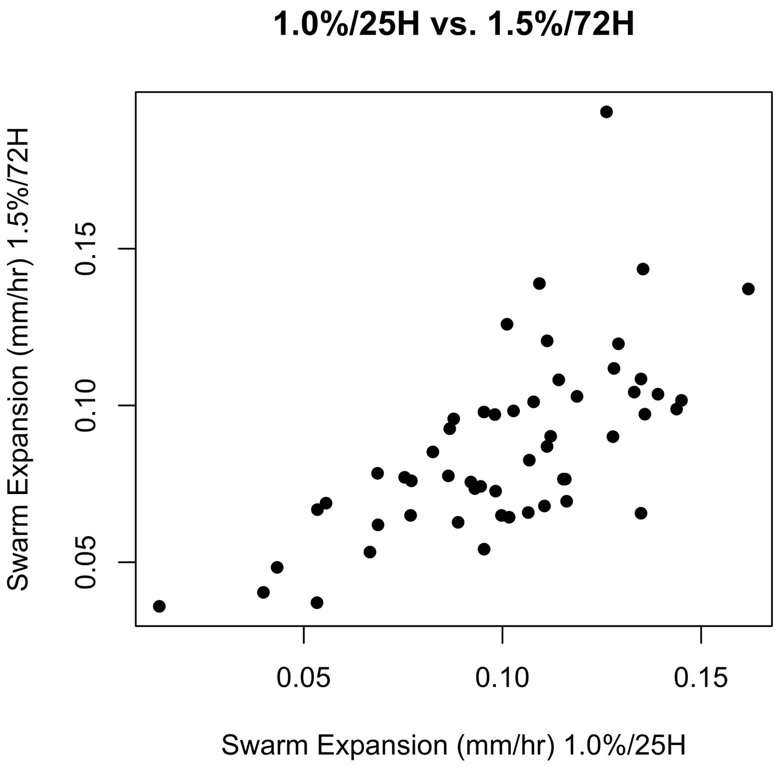
1.0%/25H vs. 1.5%/72H
Swarm Expansion (mm/hr) 1.5%/72H
0.05
0.10
0.15
Swarm Expansion (mm/hr) 1.0%/25H

E

1.0%/25H vs. Prey/25H

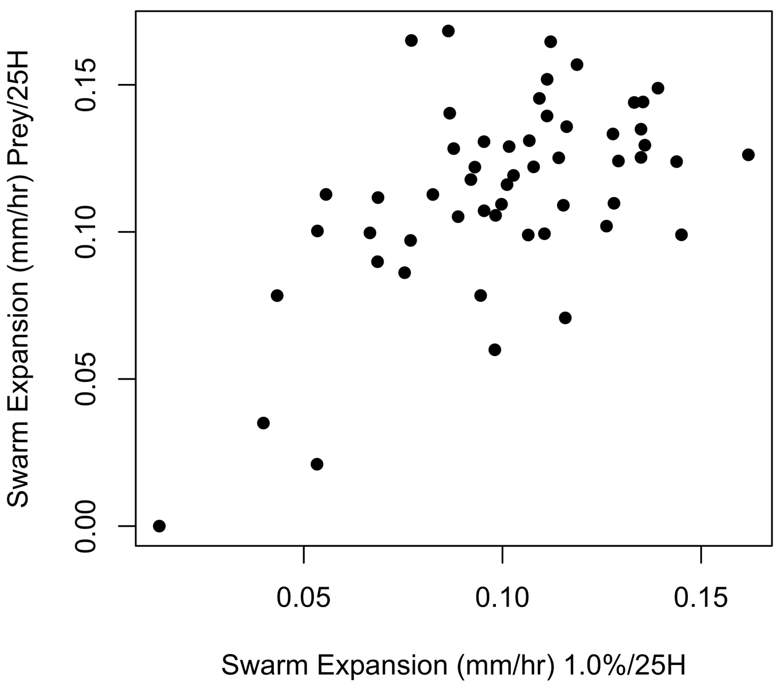

1.5%/25H vs. 0.4%/72H

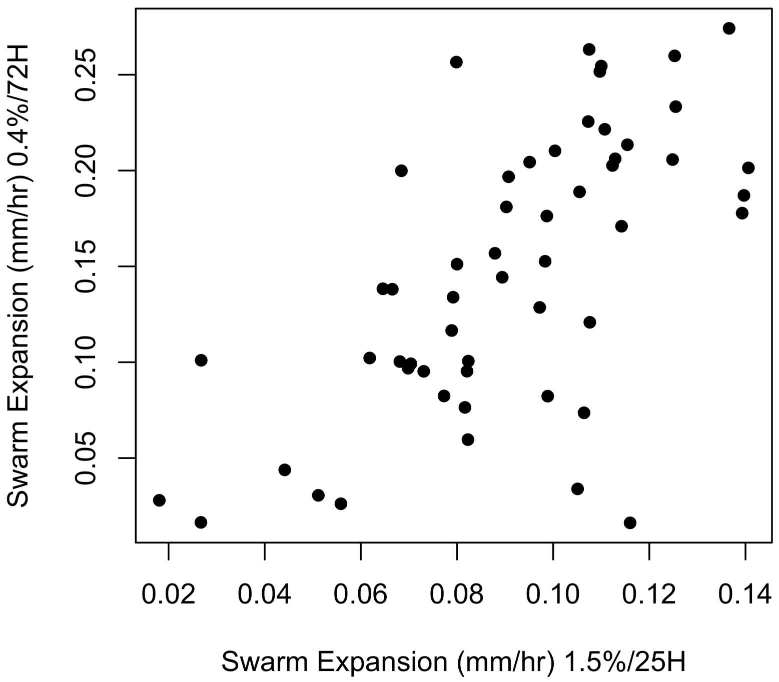

F

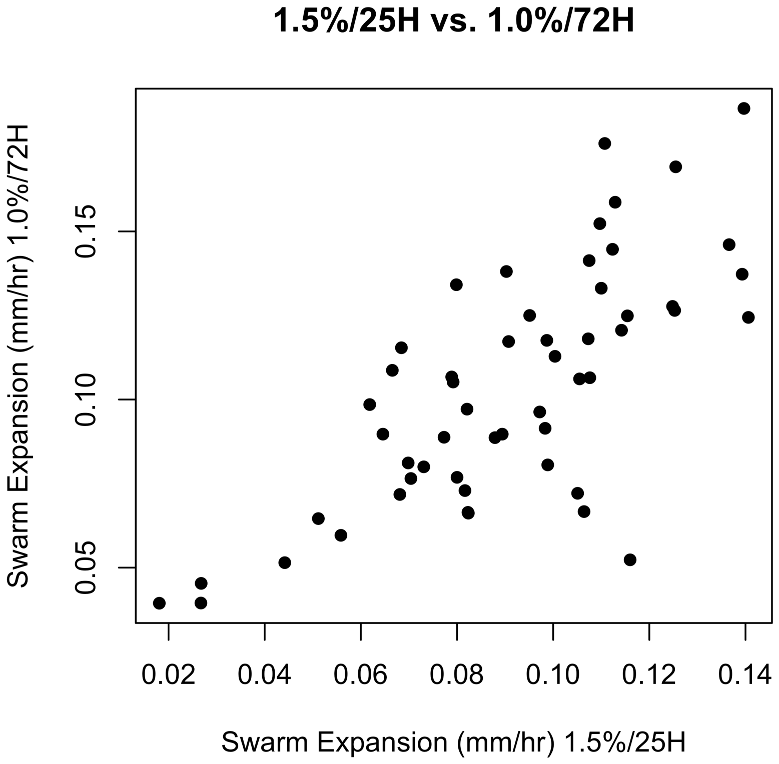

1.5%/25H vs. 1.0%/72H
Swarm Expansion (mm/hr) 1.0%/72H
0.05
0.10
0.15
0.02
0.04
0.06
0.08
0.10
0.12
0.14
Swarm Expansion (mm/hr) 1.5%/25H

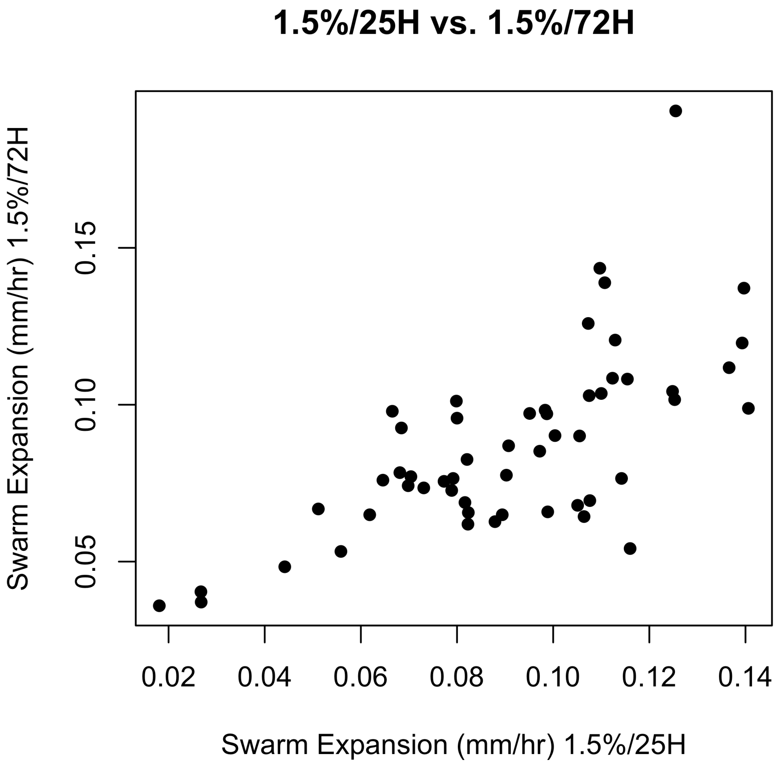

1.5%/25H vs. 1.5%/72H
Swarm Expansion (mm/hr) 1.5%/72H
0.05
0.10
0.15
0.02
0.04
0.06
0.08
0.10
0.12
0.14
Swarm Expansion (mm/hr) 1.5%/25H

G

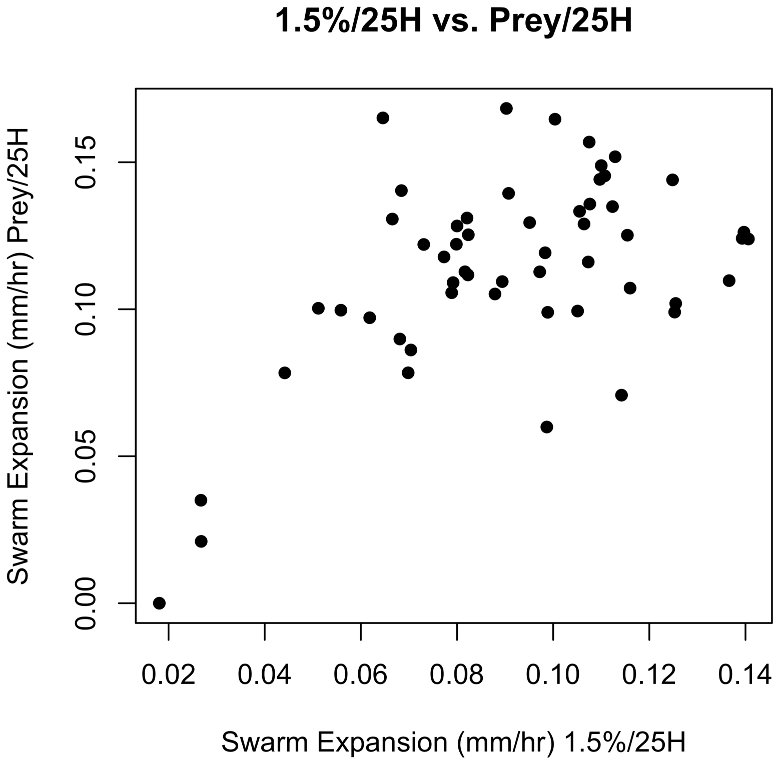

1.5%/25H vs. Prey/25H
Swarm Expansion (mm/hr) Prey/25H
0.00
0.05
0.10
0.15
0.02
0.04
0.06
0.08
0.10
0.12
0.14
Swarm Expansion (mm/hr) 1.5%/25H

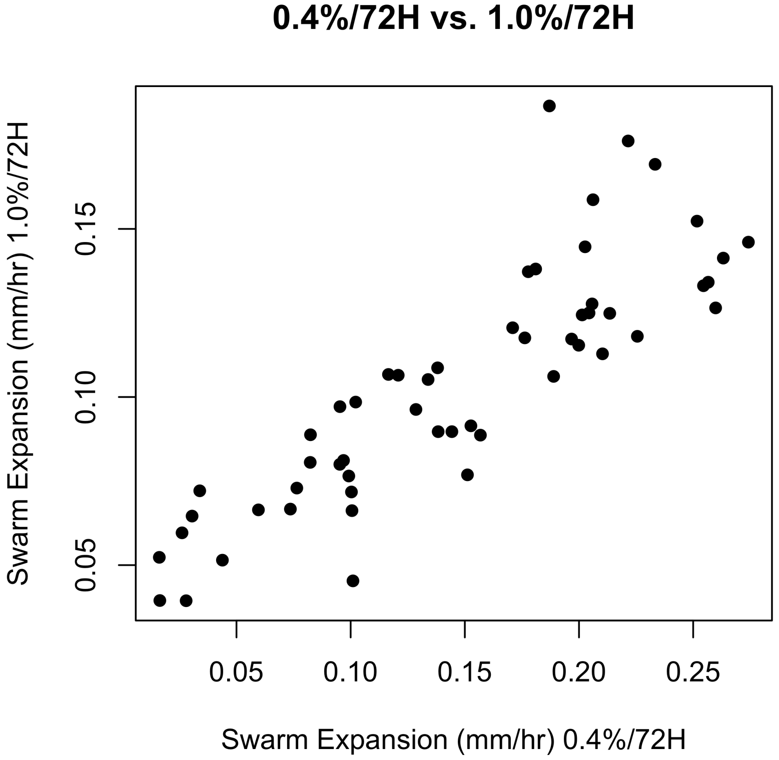

0.4%/72H vs. 1.0%/72H
Swarm Expansion (mm/hr) 1.0%/72H
0.05
0.10
0.15
0.05
0.10
0.15
0.20
0.25
Swarm Expansion (mm/hr) 0.4%/72H

H

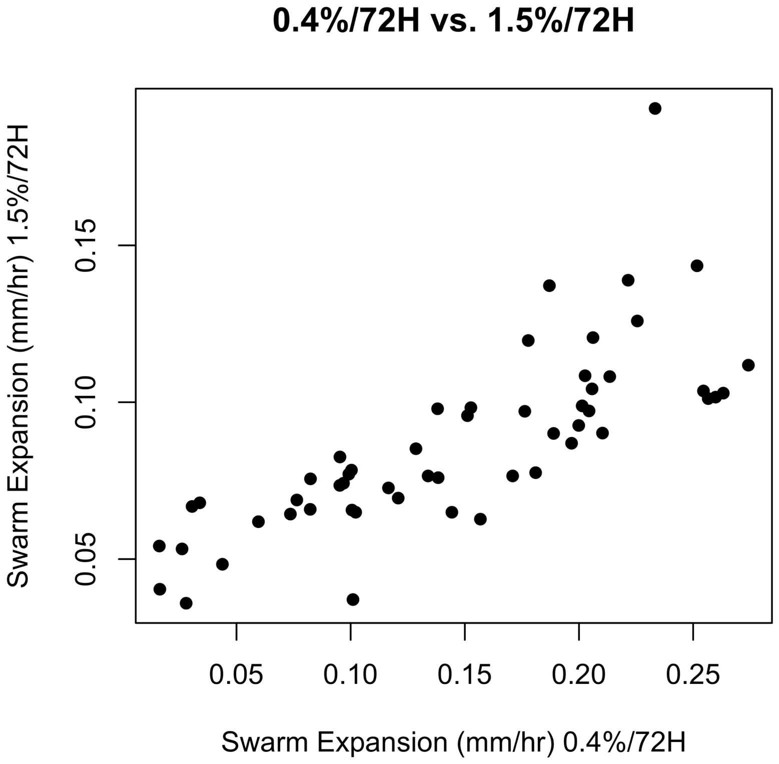
0.4%/72H vs. 1.5%/72H
Swarm Expansion (mm/hr) 1.5%/72H
0.05
0.10
0.15
0.05
0.10
0.15
0.20
0.25
Swarm Expansion (mm/hr) 0.4%/72H

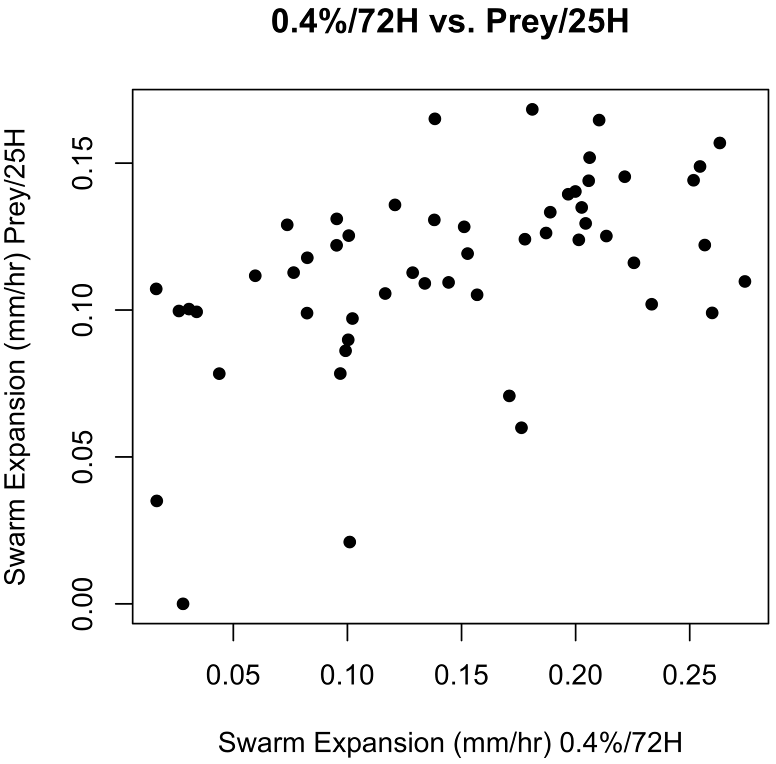
0.4%/72H vs. Prey/25H
Swarm Expansion (mm/hr) Prey/25H
0.00
0.05
0.10
0.15
0.05
0.10
0.15
0.20
0.25
Swarm Expansion (mm/hr) 0.4%/72H

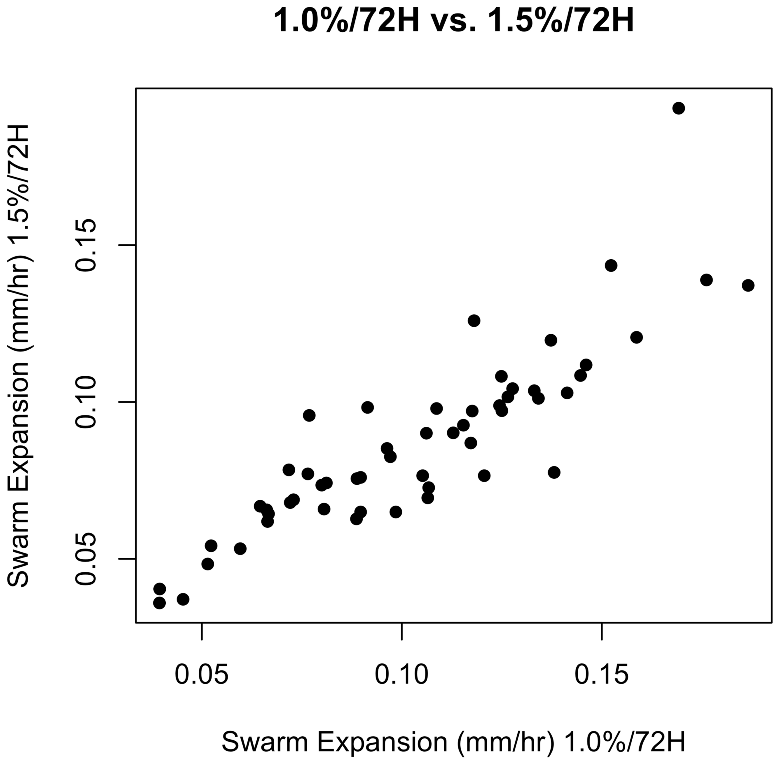
1.0%/72H vs. 1.5%/72H
Swarm Expansion (mm/hr) 1.5%/72H
0.05
0.10
0.15
Swarm Expansion (mm/hr) 1.0%/72H

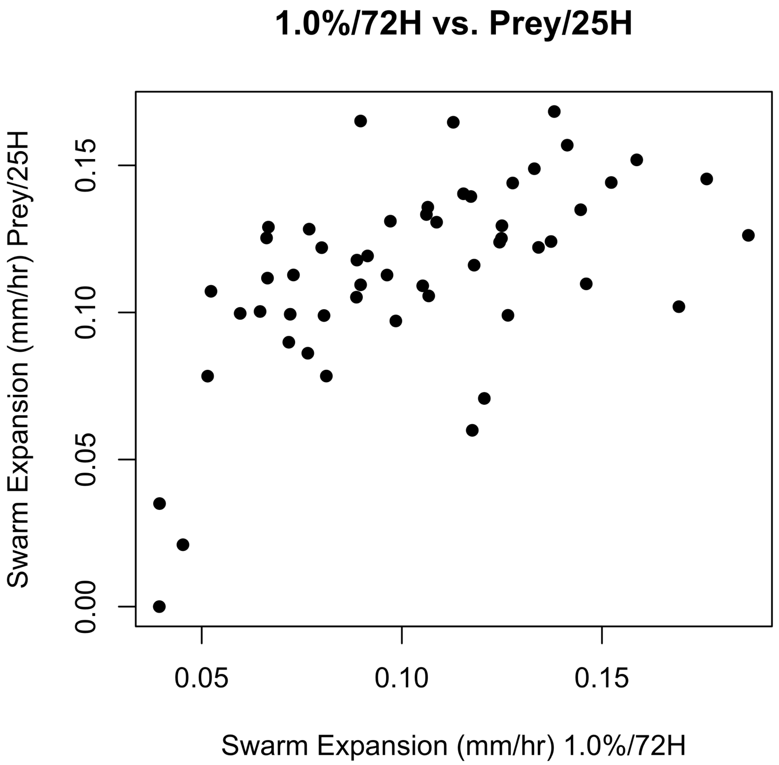
1.0%/72H vs. Prey/25H
Swarm Expansion (mm/hr) Prey/25H
0.00
0.05
0.10
0.15
Swarm Expansion (mm/hr) 1.0%/72H

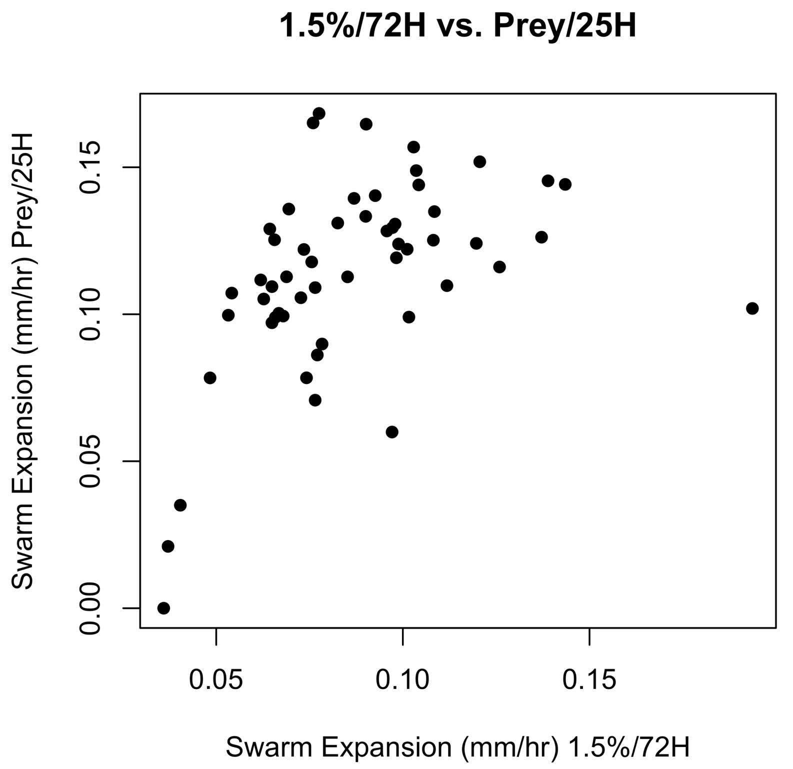
1.5%/72H vs. Prey/25H
Swarm Expansion (mm/hr) Prey/25H
0.00
0.05
0.10
0.15
0.05
0.10
0.15
Swarm Expansion (mm/hr) 1.5%/72H

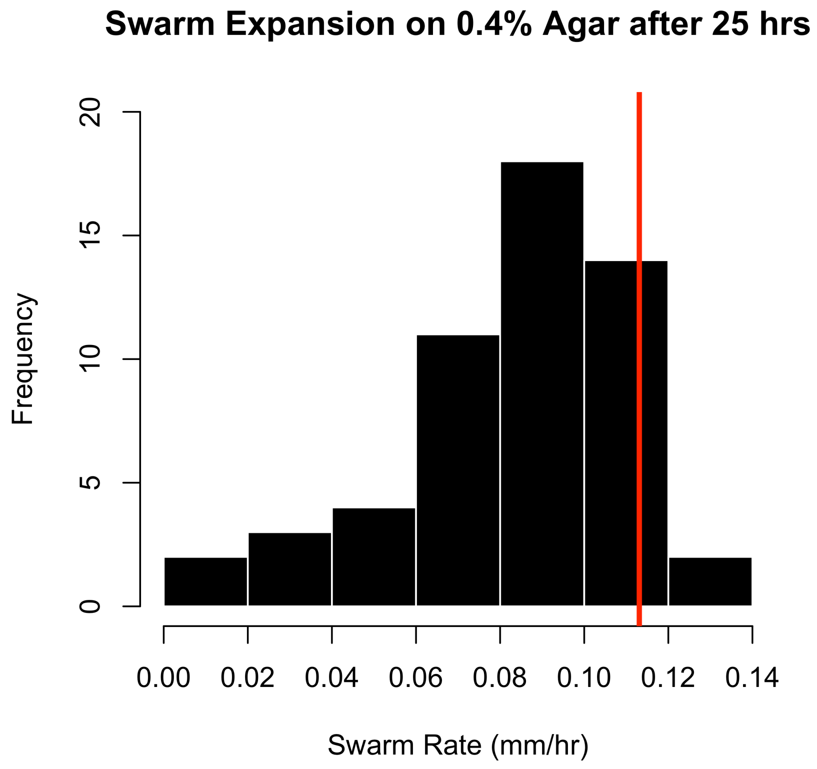
Swarm Expansion on 0.4% Agar after 25 hrs
Frequency
0
5
10
15
20
0.00
0.02
0.04
0.06
0.08
0.10
0.12
0.14
Swarm Rate (mm/hr)

K

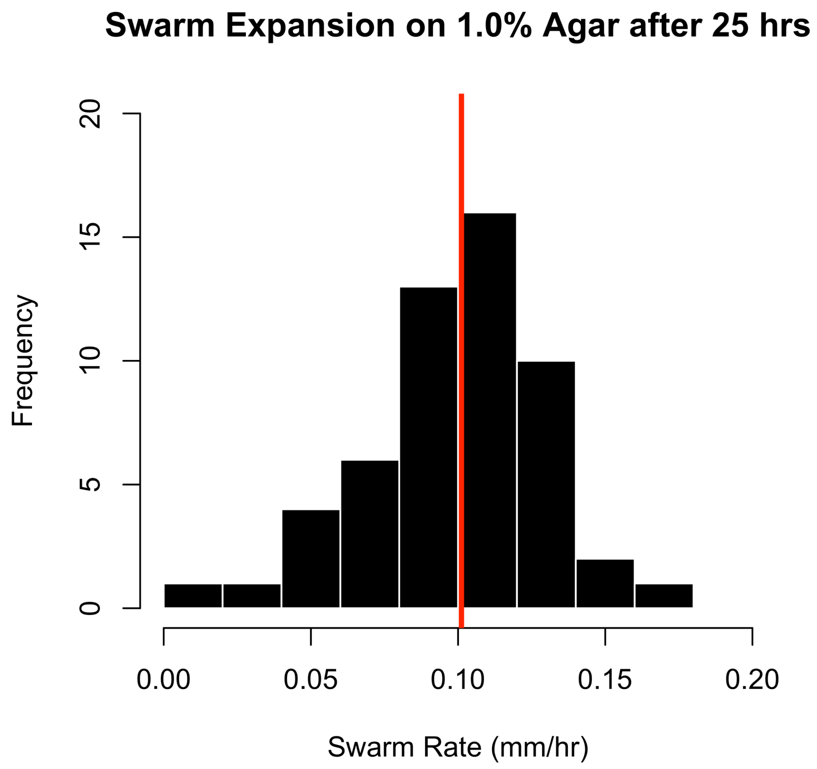

Swarm Expansion on 1.0% Agar after 25 hrs
Frequency
0
5
10
15
20
0.00
0.05
0.10
0.15
0.20
Swarm Rate (mm/hr)

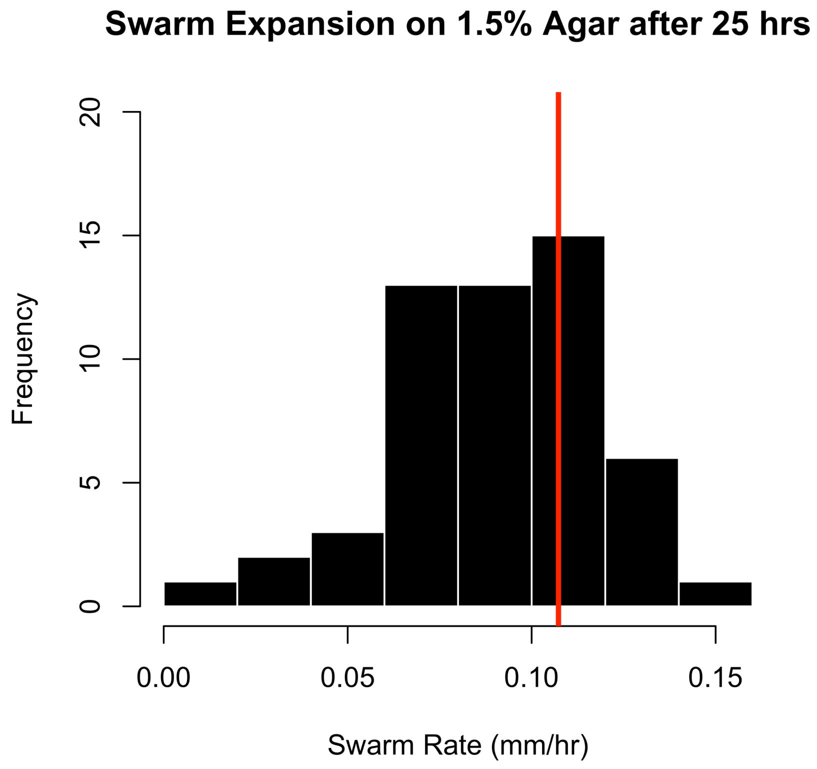

Swarm Expansion on 1.5% Agar after 25 hrs
Frequency
0
5
10
15
20
0.00
0.05
0.10
0.15
Swarm Rate (mm/hr)

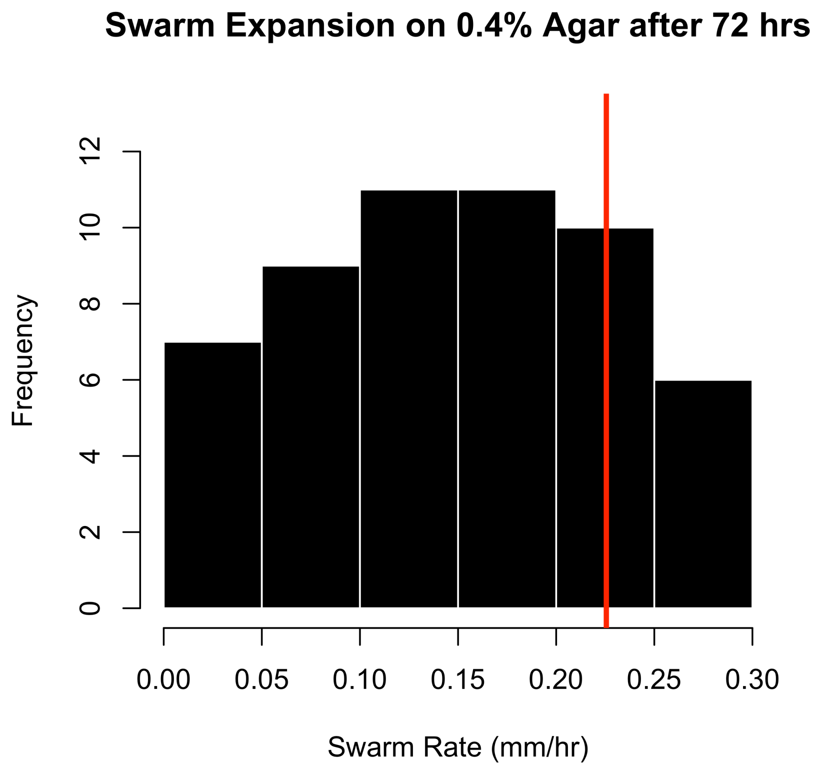
Swarm Expansion on 0.4% Agar after 72 hrs
Frequency
0
2
4
6
8
10
12
0.00
0.05
0.10
0.15
0.20
0.25
0.30
Swarm Rate (mm/hr)

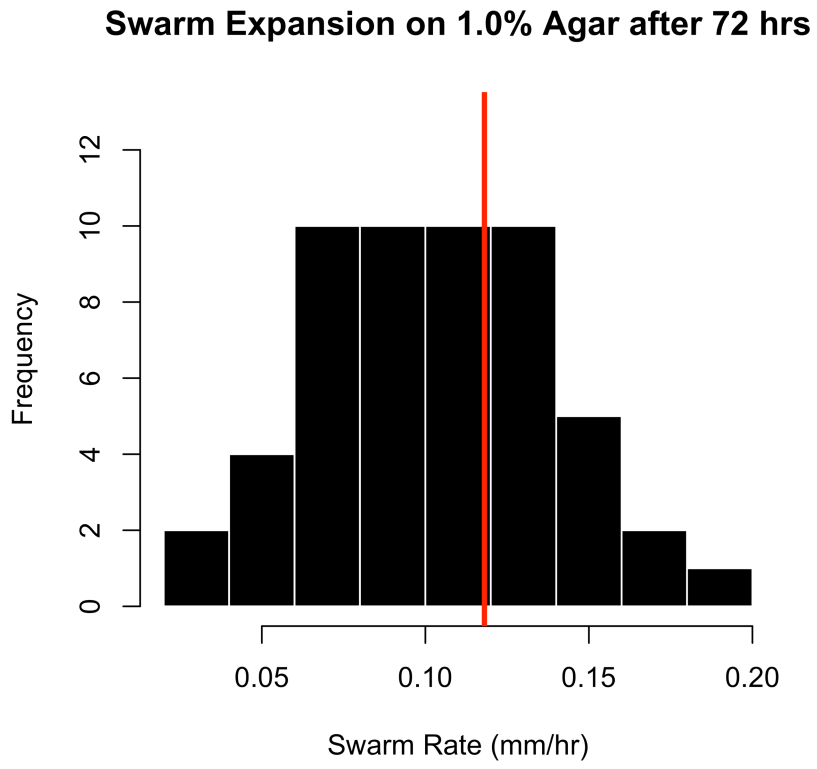
Swarm Expansion on 1.0% Agar after 72 hrs
Frequency
0
2
4
6
8
10
12
0.05
0.10
0.15
0.20
Swarm Rate (mm/hr)

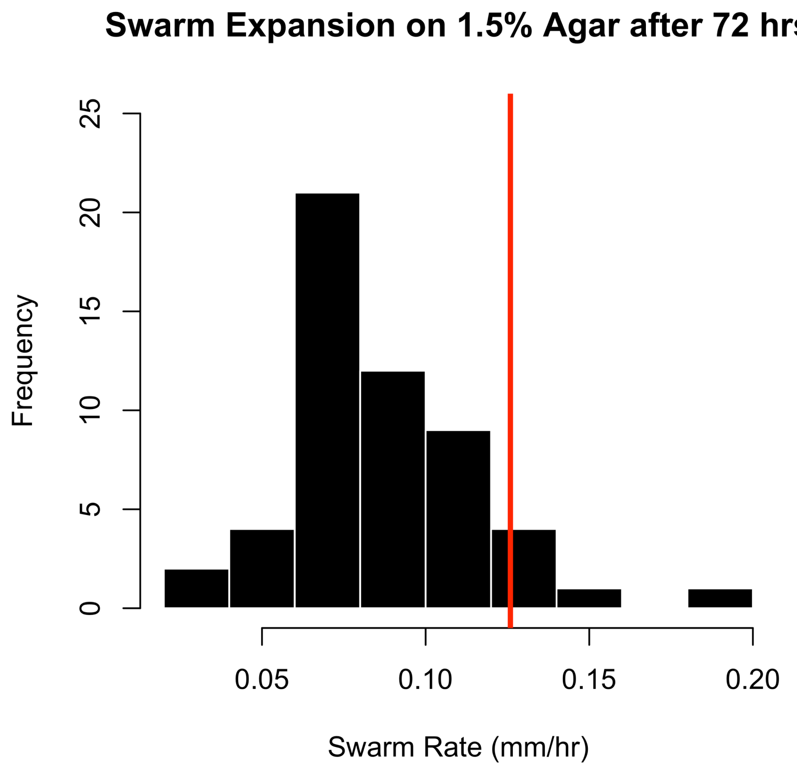
Swarm Expansion on 1.5% Agar after 72 hrs
Frequency
0
5
10
15
20
25
0.05
0.10
0.15
0.20
Swarm Rate (mm/hr)

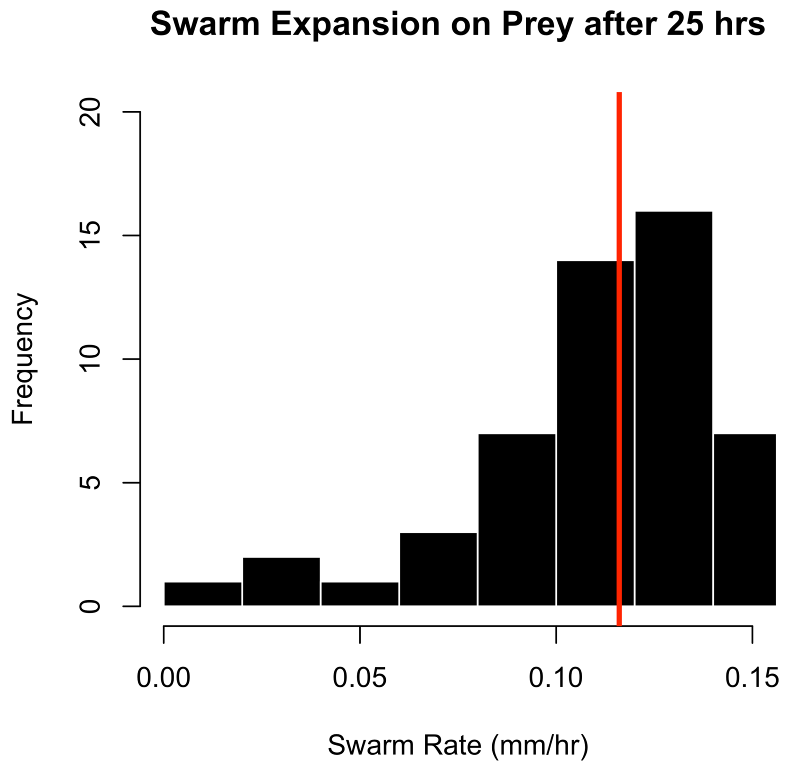
Swarm Expansion on Prey after 25 hrs
Frequency
0
5
10
15
20
0.00
0.05
0.10
0.15
Swarm Rate (mm/hr)

N
